# MicroRNAs Suppress NB Domain Genes in Tomato That Confer Resistance to *Fusarium oxysporum*

**DOI:** 10.1371/journal.ppat.1004464

**Published:** 2014-10-16

**Authors:** Shouqiang Ouyang, Gyungsoon Park, Hagop S. Atamian, Cliff S. Han, Jason E. Stajich, Isgouhi Kaloshian, Katherine A. Borkovich

**Affiliations:** 1 Department of Plant Pathology and Microbiology, Institute for Integrative Genome Biology, University of California, Riverside, Riverside, California, United States of America; 2 Department of Nematology, Institute for Integrative Genome Biology, University of California, Riverside, Riverside, California, United States of America; 3 Bioscience Division, MS M888, Los Alamos National Laboratory, Los Alamos, New Mexico, United States of America; Purdue University, United States of America

## Abstract

MicroRNAs (miRNAs) suppress the transcriptional and post-transcriptional expression of genes in plants. Several miRNA families target genes encoding nucleotide-binding site–leucine-rich repeat (NB-LRR) plant innate immune receptors. The fungus *Fusarium oxysporum* f. sp. *lycopersici* causes vascular wilt disease in tomato. We explored a role for miRNAs in tomato defense against *F. oxysporum* using comparative miRNA profiling of susceptible (Moneymaker) and resistant (Motelle) tomato cultivars. slmiR482f and slmiR5300 were repressed during infection of Motelle with *F. oxysporum*. Two predicted mRNA targets each of slmiR482f and slmiR5300 exhibited increased expression in Motelle and the ability of these four targets to be regulated by the miRNAs was confirmed by co-expression in *Nicotiana benthamiana*. Silencing of the targets in the resistant Motelle cultivar revealed a role in fungal resistance for all four genes. All four targets encode proteins with full or partial nucleotide-binding (NB) domains. One slmiR5300 target corresponds to *tm-2*, a susceptible allele of the *Tomato Mosaic Virus* resistance gene, supporting functions in immunity to a fungal pathogen. The observation that none of the targets correspond to *I-2*, the only known resistance (*R*) gene for *F. oxysporum* in tomato, supports roles for additional *R* genes in the immune response. Taken together, our findings suggest that Moneymaker is highly susceptible because its potential resistance is insufficiently expressed due to the action of miRNAs.

## Author Summary

*Fusarium oxysporum* is a fungal pathogen that represents a species complex, with members that infect numerous crops. In spite of its importance to agriculture, very little is known about roles of small RNAs in plant immunity against *F. oxysporum*. In this study, we set up a screen for tomato microRNAs (miRNAs) that correlate with resistance to *F. oxysporum f.sp. lycopersici* by performing deep sequencing of small RNAs from a resistant and susceptible tomato cultivar. We focused on two miRNAs that are uniquely down-regulated in the resistant cultivar during fungal infection. All predicted targets of these miRNAs encode proteins with NB domains, a motif associated with pathogen resistance in plants. Using a heterologous system, we validated that the miRNAs could regulate expression of four targets. Silencing of the target genes in tomato resulted in decreased immunity to *F. oxysporum* in the normally resistant cultivar. The finding that none of our targets correspond to *I-2*, the only known resistance (*R*) gene for *F. oxysporum* in tomato, supports roles for additional *R* genes in the immune response. Our results suggest that the potential resistance of the susceptible cultivar is insufficiently expressed due to the action of miRNAs.

## Introduction

MicroRNAs (miRNAs) are single-stranded RNA molecules of approximately 20–24 nucleotides in length that are endogenously transcribed from single-stranded non-coding RNA species [1], [2]. Plant miRNAs were first identified in 2002 [1], [3] and have been shown to play vital roles in multiple biological processes, including leaf morphogenesis and polarity, floral organ identity, hormone signaling and stress responses [4], [5], [6], [7], [8], [9], [10], [11]. miRNAs primarily act on their target mRNAs by influencing mRNA degradation or translational inhibition. In contrast to animals, plant mRNAs are not deadenylated prior to miRNA-guided transcript cleavage and degradation. Although there are several examples of translational inhibition of mRNAs by miRNAs in animals [12], [13], this phenomenon has only recently been reported in plants [14].

Expression of miRNA genes is regulated by external stimuli, including abiotic (e.g., drought, temperature, salinity) and biotic (e.g., pathogens such as viruses, bacteria and fungi) stresses. During pathogen attack, recognition of microbe-associated molecular patterns (MAMPs) by plant pattern-recognition receptors leads to pattern-triggered immunity (PTI) resulting in changes in gene expression that result in altered hormone and metabolite levels [15]. Pathogens have evolved effectors to sabotage PTI. In return, plants acquired disease resistance (*R*) genes, to recognize the presence or action of specific effectors, directly or indirectly, and to activate effector-triggered immunity (ETI), a fast and strong form of immunity [16].

A role for miRNAs in regulating genes important for plant defense has been demonstrated for the response to several pathogens [17]. In tomato (*Solanum lycopersicum*), the levels of miR319/miR159 and miR172 are induced during *Tomato leaf curl New Delhi virus* (ToLCNDV) disease progression [18]. miR393, miR160 and miR167 are up-regulated in leaves challenged with the virulent bacterial pathogen *Pseudomonas syringae* pv. *tomato* (*Pst*) DC3000 [19]. Similarly, miR393, miR319, miR158, miR160, miR167, miR165/166 and miR159 are induced, while miR390, miR408 and miR398 are repressed, in *Arabidopsis thaliana* (Arabidopsis) leaves infected with *Pst* DC3000 [20]. Treating Arabidopsis with the MAMP flagellin-derived peptide, flg-22, induces expression of miR393, a negative regulator of mRNAs for the F-box auxin receptors TIR1, AFB2, and AFB3 [21]. miR482a, a member of the miR482/2118 superfamily, targets mRNAs for R proteins, with nucleotide-binding site (NB) and leucine-rich repeat (LRR) motifs, for degradation both directly and through generation of secondary small interfering RNAs (siRNAs) in *Nicotiana benthamiana* infected with *Pst* DC3000 [22], [23]. miR5300 was first identified as a novel tomato miRNA [24] and later classified as a member of the miR482/2118 superfamily [23]. However, regulation of predicted target genes by miR5300 has not yet been reported [25].

Strains of the ascomycete fungus *Fusarium oxysporum* are ubiquitous soil inhabitants [26], [27]. Accumulating data indicate that *F. oxysporum* is a large species complex, with more than 120 *formae speciales* causing disease in vegetables, fruit trees, wheat, corn, cotton and ornamental crops [26], [27]. *F. oxysporum* infects vascular bundles in the plant host, leading to wilt symptoms. Germination of dormant spores in soil results in adherence and invasion of plant roots by fungal hyphae. The hyphae then move from the root cortex to the xylem where production and dissemination of microconidia spores is critical for disease progression [26].

Previous work has demonstrated that the *I-2* gene of tomato confers resistance to race 2 strains of *F. oxysporum* f. sp. *lycopersici* (hereafter referred to as *F. oxysporum*; [28]). The *I-2* locus encodes a coiled-coil (CC) NB-LRR protein that recognizes the *avr2* gene product from *F. oxysporum* [29]. The near-isogenic tomato cultivars Moneymaker and Motelle are susceptible (*i-2/i-2*) and resistant (*I-2/I-2*) genotypes, respectively, for *I-2* and the response to *F. oxysporum* infection [30], [31], [32].

In this study, we explored a possible role for tomato miRNAs in the differential resistance of Moneymaker and Motelle to *F. oxysporum*. Our results indicate that two different miRNAs contribute to plant immunity in tomato by influencing mRNA stability or translation of at least three NB domain-containing proteins distinct from I-2.

## Results

### Identification of microRNAs induced by *F. oxysporum* in tomato roots

We investigated microRNA (miRNA) production in roots of tomato during infection with the wilt fungus *F. oxysporum* through construction of small RNA libraries and deep sequencing. We took advantage of two near-isogenic cultivars that show differential interaction with *F. oxysporum* – Moneymaker (susceptible) and Motelle (resistant) [30], [31], [32]. We generated a total of four libraries, including: Moneymaker treated with water (MM_H_2_O), Moneymaker treated with *F. oxysporum* (MM_Foxy), Motelle treated with water (Mot_H_2_O) and Motelle treated with *F. oxysporum* (Mot_Foxy). Our goal was to identify miRNAs that were either upregulated in Moneymaker or down-regulated in Motelle after infection with *F. oxysporum*. Such a pattern of expression would presumably lead to upregulation of potential target mRNAs required for plant defense in Motelle, but not Moneymaker, after infection.

**Figure 1:**
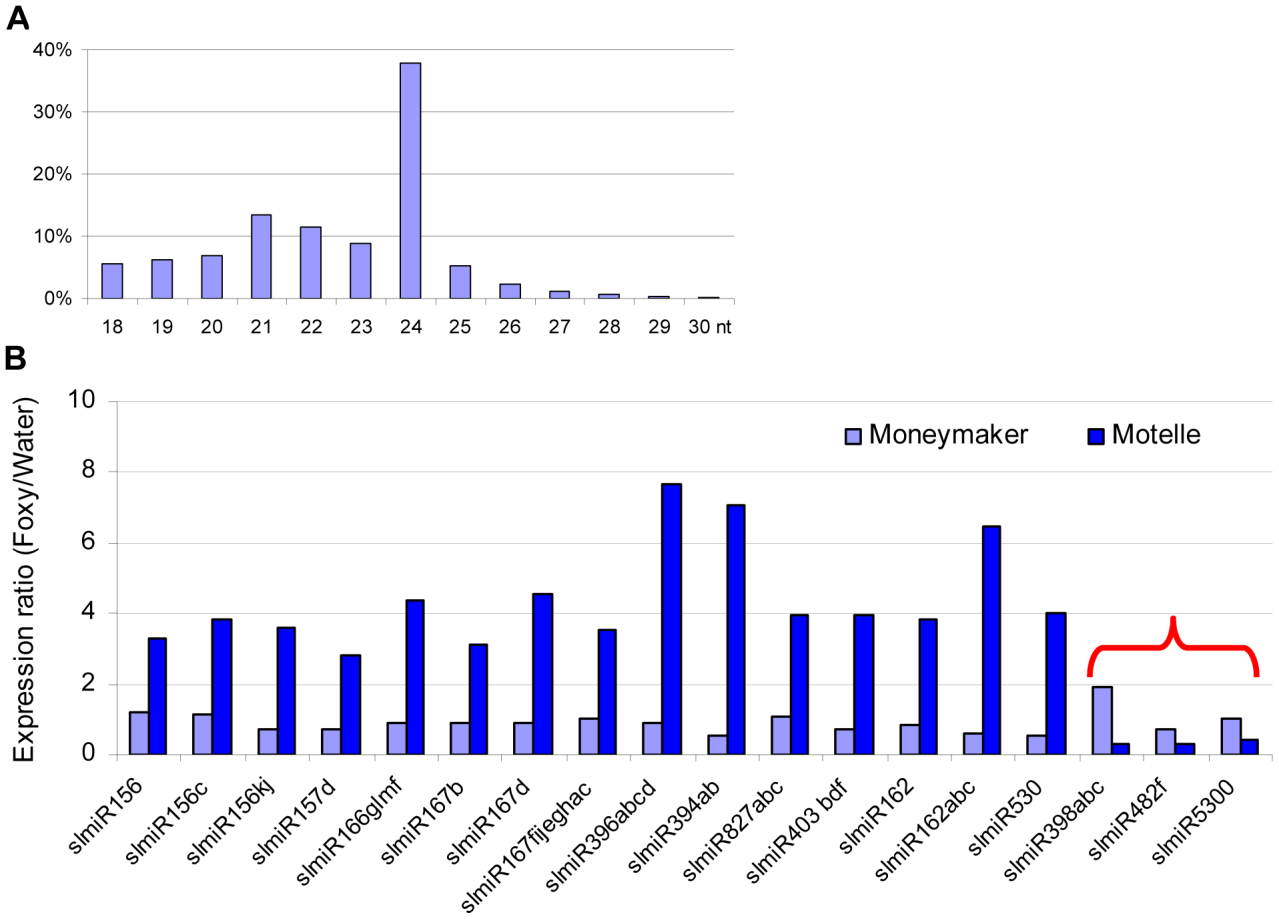
Properties of miRNAs expressed in resistant and susceptible tomato cultivars treated with water or the fungal pathogen *Fusarium oxysporum*. In total, four small RNA libraries were subjected to deep sequencing: susceptible tomato cultivar Moneymaker treated with water; Moneymaker treated with *Fusarium oxysporum f. sp. lycopersici* (*F. oxysporum*); resistant tomato cultivar Motelle treated with water and Motelle treated with *F. oxysporum*. The sequence length of small RNAs (**A**) for the combined data from the four libraries was determined as described in the Materials and Methods. Relative expression levels of known miRNAs (**B**) were determined by dividing normalized reads for *F. oxysporum* treatment by those for water treatment for each cultivar. The red bracket indicates the miRNAs that were negatively regulated in Motelle, but not Moneymaker, after *F. oxysporum* infection. Putative roles/targets of miRNAs in various plant species (information from miRBase.org): miR156, miR156c, miR156kj and miR157d: Squamosa-promoter Binding Protein (SBP)-like transcription factors; miR166glmf: HD-Zip transcription factors, including Phabulosa (PHB) and Phavoluta (PHV) that regulate axillary meristem initiation and leaf development; miR167b, miR167d and miR167fijeghac: Auxin Response Factors (ARF transcription factors); miR396abcd: Growth Regulating Factor (GRF) transcription factors, rhodenase-like proteins, and kinesin-like protein B; miR394ab, F-box proteins; miR827abc: Unknown; miR403bdf: Virus defense; miR162 and miR162abc: Unknown; miR530: Unknown; miR398abc: copper superoxide dismutases and cytochrome C oxidase subunit V; miR482f: NB domain proteins; miR5300, Unknown.

Using Illumina sequencing, we obtained a total of more than 27 million high quality small RNA sequences from the four libraries that could be mapped to the tomato genome. Of these, 5,743,067 were from MM_H_2_O, 5,492,955 from MM_Foxy, 4,392,583 from Mot_H_2_O and 5,497,730 from Mot_Foxy (Table S1). Among all size classes, 24-, 21- and 22-nt small RNA species were the three most abundant (Fig. 1A). These sizes are similar to those previously identified in tomato [22], [23]. Within the miRNA population of sequences, more than 98% of the reads began with a uracil. It has been demonstrated that Argonuate proteins recruit small RNAs based on the 5′ terminal nucleotide: AGO2 and AGO4 recruit small RNAs with 5′ terminal adenosine, whereas AGO1 and AGO5 recruit small RNAs with a 5′ terminal uracil and cytosine, respectively [33], [34], [35]. We identified 82 predicted miRNAs with at least one raw sequence read in one of the four libraries (Table S2). miRNAs were considered for further analysis if there were at least 12 raw sequence reads and at least a two-fold change between the *F. oxysporum*-infected and control plant libraries. Based on these criteria, we identified 18 unique miRNA sequences corresponding to plant disease resistance, stress responses, transcription factors, and others (Fig. 1B). Notably, among all of the regulated miRNAs identified, miR403 and miR398 are associated with disease resistance in other plant species [36], [37]. miR398 is also implicated in the regulatory network for additional abiotic stresses, including salinity, water deficit, oxidative stress, high levels of abscisic acid, ultraviolet light, copper and phosphate deficiency and high sucrose [11], [38], [39], [40], [41], [42], [43], [44]. In contrast to the other regulated miRNAs, functions for miR5300 have not been previously reported in any plant species, including tomato.

As stated above, our objective in this study was to identify miRNAs that were present at increased levels in Moneymaker or decreased levels in Motelle after infection with *F. oxysporum*. Our results showed that the majority of miRNAs (15) were present at increased levels in Motelle plants after infection with *F. oxysporum* and we did not identify any miRNAs that were increased (or decreased) at least two-fold in Moneymaker after infection (Fig. 1B). In contrast, slmiR398, slmiR5300 and slmiR482f were all suppressed at least two-fold in Motelle plants after *F. oxysporum* treatment (Fig. 1B), consistent with our original hypothesis.

**Figure 2:**
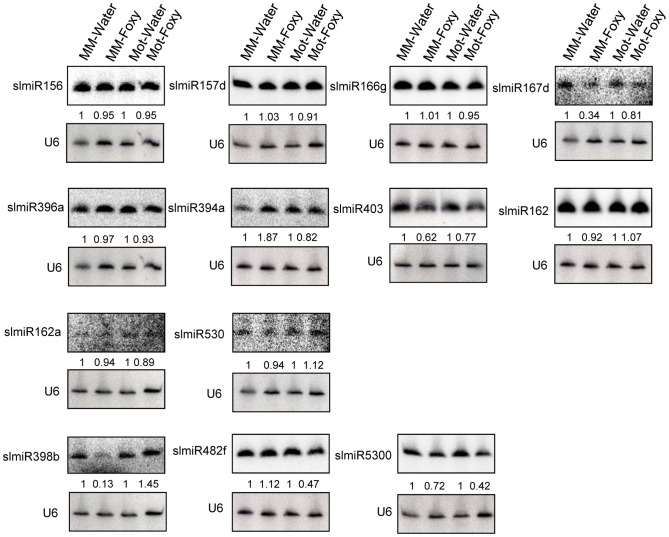
Northern blot analysis of miRNAs. Oligonucleotide probes were used to quantitate levels of several miRNAs identified during deep sequencing using northern blot analysis. Root total RNA samples (40 µg each) were from Moneymaker treated with water (MM-H_2_O), Moneymaker infected with *F. oxysporum* (MM-Foxy), Motelle treated with water (Mot-H_2_O) and Motelle infected with *F. oxysporum* (Mot-Foxy). U6 RNA served as a loading control for each blot. Blots were imaged using a Phosphorimager and miRNA species quantitated using Imagequant software, with normalization to the amount of U6 RNA. The numbers below each blot indicate the amount of miRNA in each sample relative to the corresponding water-treated control. Note that due to significant sequence homology, slmiR482 subfamily members cannot be quantitatively distinguished from one another using northern analysis.

Northern blot analysis was performed to analyze expression of the three miRNAs that were demonstrated to be down-regulated in Motelle by deep sequencing. We analyzed 14 additional miRNAs (17 total) in order to avoid excluding other possible candidates due to issues with sequencing data. The 13 miRNAs that could be detected using northern analysis are presented in Fig. 2. A subset of the small RNA northern blot results was consistent with the deep sequencing data. A caveat to this analysis is that subfamily members (e.g., slmiR482a-f; [23], [45], [46], [47] that share significant homology will cross-hybridize during this analysis. Of interest, both slmiR482f and slmiR5300 were decreased in Motelle plants treated with *F. oxysporum* as detected by both methods (Fig. 1B and Fig. 2). The reduction observed during northern analysis (Fig. 2; 53% for slmiR482f and 58% for slmiR5300) was similar to that obtained during deep sequencing (Table S2; 72% for slmiR482f and 61% for slmiR5300). Deep sequencing data showed that slmiR398 was induced by 1.89-fold in Moneymaker by *F. oxysporum* infection, but suppressed by 71% in Motelle (Table S2). However, these expression trends were essentially reversed in the small RNA northern blot analysis. slmiR398 levels were similar in Moneymaker and Motelle controls, elevated in Motelle treated with *F. oxysporum* and barely detectable in Moneymaker under the same conditions (Fig. 2). Thus, the northern results for slmiR398 were reversed relative to those from deep sequencing for infected Moneymaker and Motelle plants. Deep sequencing data indicated that expression of slmiR403 was reduced by 26% in Moneymaker plants, but induced four-fold in Motelle, after treatment with *F. oxysporum* (Fig. 1B). Although the small RNA northern results also detected slight reduction of slmiR403 in Moneymaker, slmiR403 levels were slightly reduced in Motelle treated with *F. oxysporum* (Fig. 2).

Based on our original hypothesis, the results from both deep-sequencing and small RNA northern blot analysis pointed to slmiR482f and slmiR5300 as potential regulators of plant defense genes in tomato. Levels of both miRNAs decreased in the resistant Motelle plants after infection with *F. oxysporum*. These two miRNAs belong to the miR482/2118 Superfamily in tomato [23], [48], [49].

### Predicted targets of slmiR482f and slmiR5300 exhibit altered expression after infection of tomato by *F. oxysporum*

**Figure 3:**
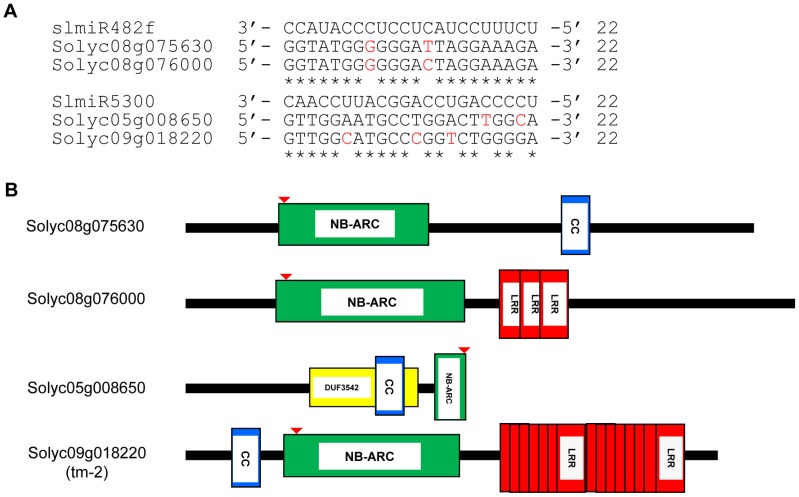
Alignment of slmiRNA sequences with predicted mRNA targets and protein domain analysis. A. **Predicted mRNA target sequences.** Targets for slmiR482f and slmiR5300 were identified using the Sol Genomics website (http://solgenomics.net). Alignments were made using ClustalW2. The nucleotides shown in red in each mRNA target are mismatches with the corresponding miRNA. B. **Protein domains.** Analysis was performed using Interpro (http://www.ebi.ac.uk/interpro/). NB: A signaling motif shared by plant resistance gene products and regulators of cell death in animals. CC: Coiled-Coil domain. LRR: Leucine-rich repeats. DUF3542: Protein domain of unknown function found in eukaryotes and viruses. The NB domain in Solyc05g008650 is truncated at the C-terminus. The red arrow indicates the miRNA binding site in the P-loop region of the NBS-ARC domain.

We utilized the psRNATarget algorithm [50] that predicts targets of plant miRNAs to identify mRNAs with binding sites for slmiR482f and slmiR5300 (See Methods). For each miRNA, we found several potential targets in the tomato genome (Fig. S1). Interestingly, all top putative targets for either miRNA encode proteins with full or partial NB domains (Fig. 3B). The binding site for both slmiR482f and slmiR5300 miRNAs is in the P-loop region of the NB domain in each target (indicated by red arrow in Fig. 3B). For slmiR482f, the top two putative targets were Solyc08g075630 (NB and CC domains) and Solyc08g076000 (NB and three LRR domains) (Fig. 3A, B). Solyc08g075630 has an atypical arrangement, with the CC domain following the NB domain (Fig. 3B). For slmiR5300, the top two putative targets were Solyc05g008650 and Solyc09g018220 (Fig. 3A,B). Solyc05g008650 contains a truncated NB domain and overlapping DUF3542 and CC motifs (Fig. 3B). We analyzed available RNAseq data, as well as all three reading frames of genomic sequence at the Sol Genomics database (http://solgenomics.net/organism/Solanum_lycopersicum/genome) downstream from this gene, but could not find sequence corresponding to the rest of the NB domain. DUF3542 is a domain of unknown function found in eukaryotes and viruses [51]. Interestingly, the CC-NB-LRR domain protein-encoding gene Solyc09g018220 is *tm-2* [52], [53]. Tomato cultivars Motelle and Moneymaker contain *tm-2* (http://tgc.ifas.ufl.edu/vol43/p79.html), the susceptible allele of the *Tm-2^2^* locus [53]. *Tm-2^2^* is required for durable resistance of tomato to *Tomato mosaic virus* (ToMV) [52].

**Figure 4:**
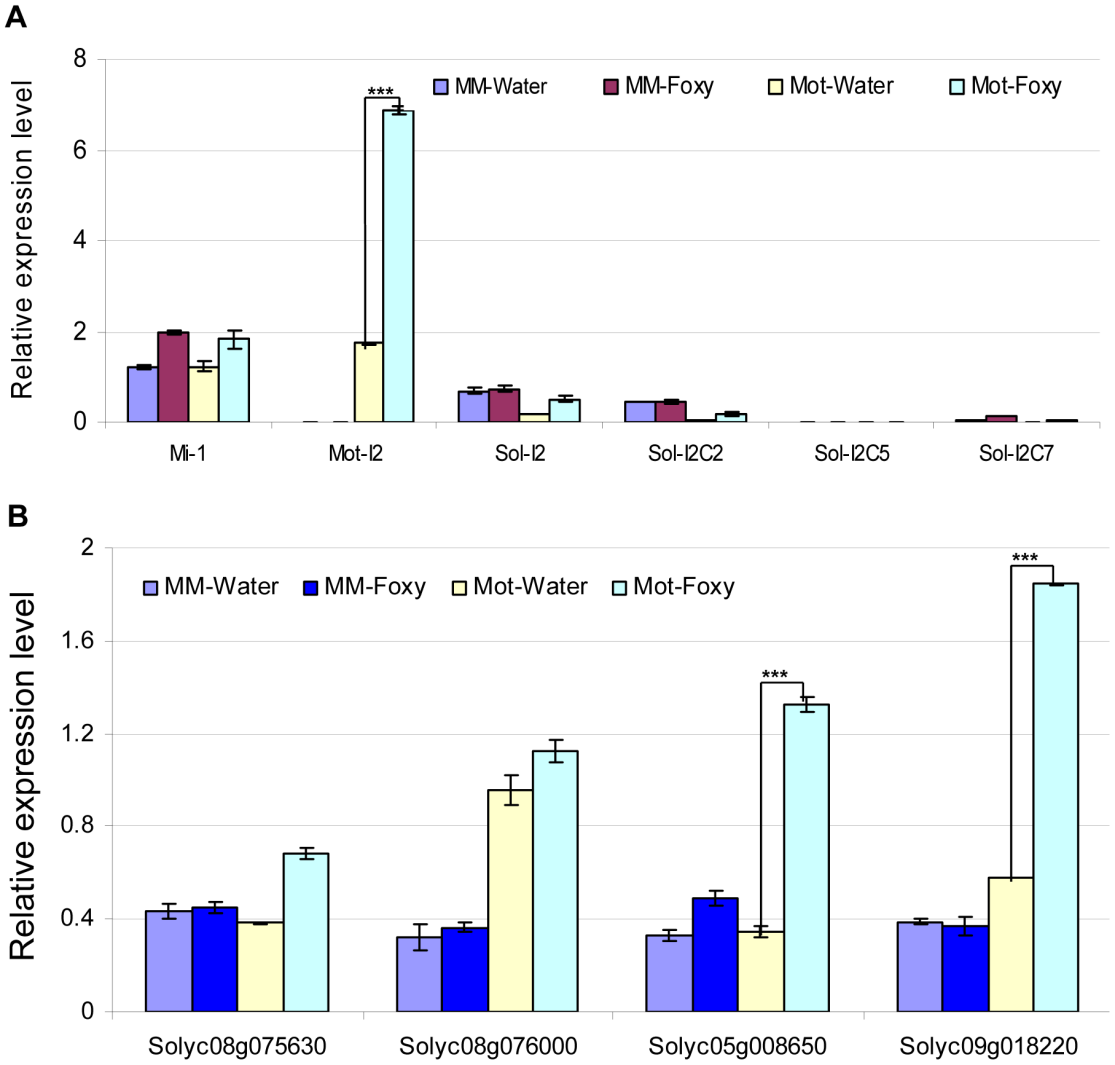
Determination of mRNA levels for control genes and predicted targets in tomato cultivars using qRT-PCR. Total tomato root RNA was used for qRT-PCR with gene-specific primers. Errors are expressed as the standard error. * and *** indicate significant differences when compared to the corresponding control plants in the same treatments at p<0.05 and p<0.001, respectively. A. **Transcript levels of** ***Mi-1*** **,** ***I-2*** **and four** ***I-2*** **homologs.** Motelle and Moneymaker have different alleles of *Mi-1* [89], while Moneymaker appears to lack *I-2*. The prefix “Mot” signifies that the indicated *I-2* homolog has been previously identified in Motelle [28], while “Sol” indicates that the homolog was found during BLAST searches of the Sol database. B. **Expression of the four predicted target genes.** Solyc08g075630 and Solyc08g076000 are predicted targets of slmiR482f, while Solyc05g008650 and Solyc08g018220 are predicted targets of slmiR5300.

We next tested the possibility that the presence of slmiR482f or slmiR5300 would suppress expression of the target genes, leading to reduced levels of the encoded mRNAs and proteins. Before quantitating expression of putative target mRNAs, we first determined the expression levels of several control genes in our four RNA preparations using qRT-PCR (Fig. 4A). These included *I-2*, required for *F. oxysporum* resistance [29], several *I-2*-homologous genes identified in the Sol Genomics database, and *Mi-1*, required for resistance to nematodes and other pests, but not *F. oxysporum*. *Mi-1* was chosen because Motelle and Moneymaker are also near-isogenic for this gene [30]. The results for *I-2* and *Mi-1* were in agreement with previous findings and the genotypes of the two cultivars [30]. *I-2* was not detectable in Moneymaker, but levels increased more than 3-fold in Motelle after infection with *F. oxysporum*. *Mi-1* levels were similar in both cultivars and did not change significantly after *F. oxysporum* treatment. Expression of the four *I-2*-homologous genes varied, but none exhibited a significant difference between water control and *F. oxysporum* exposure.

We checked the mRNA levels of the putative targets under water or *F. oxysporum* treatment conditions in both tomato cultivars using qRT-PCR (Fig. 4B) and northern blot analysis (Fig. S2). The results of qRT-PCR showed that putative slmiR482f target Solyc08g075630 was induced by almost two-fold in Motelle, but unchanged in Moneymaker, after treatment with *F. oxysporum* (Fig. 4B). The results from northern analysis of Solyc08g075630 closely mirrored those from qRT-PCR (Fig. S2). Both qRT-PCR and northern analysis demonstrated that Solyc08g076000 mRNA levels were not significantly changed by *F. oxysporum* treatment in either cultivar, although levels of Solyc08g076000 were elevated in Motelle relative to Moneymaker (Fig. 4B, Fig. S2). qRT-PCR and northern analysis revealed significant upregulation of slmiR5300 putative target Solyc05g008650 in Motelle (3–4 fold), but not Moneymaker, after infection with *F. oxysporum* (Fig. 4B, Fig. S2). Likewise, slmiR5300 target Solyc09g018220 exhibited 3.4- (Fig. 4B) or 5.9-fold (Fig. S2) up-regulation by *F. oxysporum* infection in Motelle compared to water, while levels in Moneymaker were unchanged. Taken together, these results support regulation of at least three of the four predicted target genes at the mRNA abundance level by their respective miRNAs.

### The four target genes are affected by slmiR482f or slmiR5300 in co-expression studies

The psRNATarget algorithm results predicted that both slmiR482f predicted targets and one slimiR5300 target (Solyc09g018220) were regulated at the translational level, while the second slmiR5300 target (Solyc05g008650) is regulated at the mRNA cleavage step. The results from our mRNA analysis were consistent with pre- or post-transcriptional regulation of certain target genes, as three targets exhibited elevated transcript levels in Motelle, but not Moneymaker, with fungal infection, while one target (Solyc08g076000) was relatively unchanged. In order to further probe the possible mechanism for regulation of targets by the four miRNAs, as well as determine specificity of the miRNA/target interaction, we conducted Agrobacterium-mediated transient co-expression experiments in *N. benthamiana*. We used a binary construct to co-express the FLAG-tagged putative target protein gene and the respective miRNA gene. The presence of the FLAG tag would allow us to detect differences in protein levels and thus, possible translational or post-translational regulation of the target by the miRNA. Vectors with no insert, only a target gene, or containing the miRNA gene slmiR166 that does not recognize our predicted targets, were used as negative controls.

**Figure 5:**
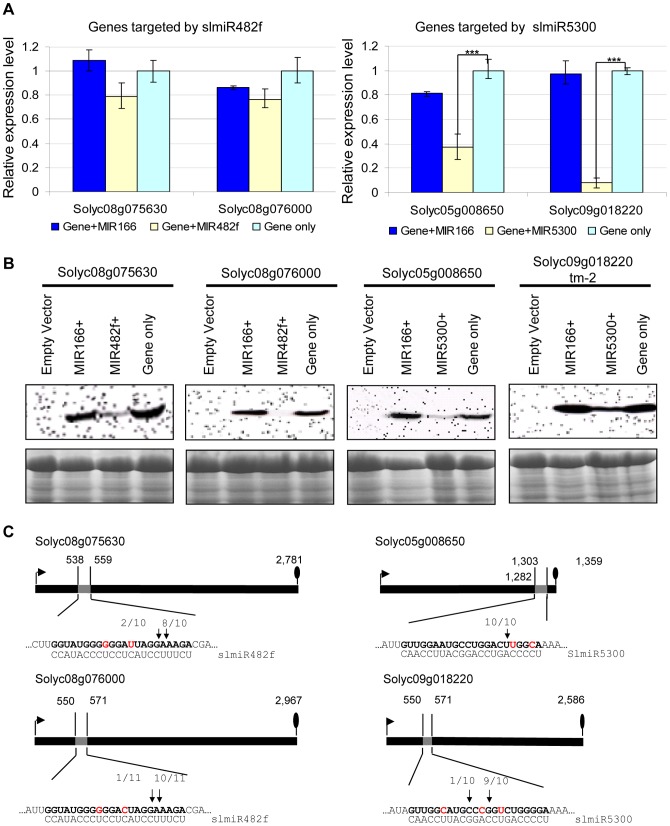
Co-expression of slmiRNAs and predicted targets in *Nicotiana benthamiana* leaves. **A. Levels of target mRNAs.** qRT-PCR was used to determine relative levels of predicted tomato mRNAs in *N. benthamiana* leaves expressing only empty vector; target mRNA and a control miRNA (slmiR166); target mRNA and the appropriate miRNA (slmiR482f or slmiR5300); or target mRNA and empty vector. Values were normalized to *N. benthamiana* actin. Errors are expressed as the standard error. *** indicates significant differences when compared to the corresponding control plants in the same treatments at p<0.001. B. **Target protein levels.** Total protein isolated from the samples in (A) was electrophoresed on SDS-PAGE gels and blotted onto nitrocellulose membranes. A FLAG antiserum was used to detect the tagged target proteins during western analysis as described in the Materials and Methods (top panels). A duplicate gel was Coomassie-stained and used as a loading control (bottom panels). Similar results were obtained for two biological replicates. C. **Diagrammatic representation of target mRNA cleavage sites determined using 5′RACE.** Thick black lines represent open reading frames (nucleotide numbering begins at the start codon indicated by the black arrow), and the putative miRNA interaction site is shown as a gray box, with the nucleotide position within the ORF indicated. The nucleotides shown in red in each mRNA target are mismatches with the corresponding miRNA. The number of sequenced 5′RACE clones corresponding to each site is indicated by vertical arrowheads.

We first performed qRT-PCR to check the mRNA levels of targets during co-expression (Fig. 5A). In the presence of slmiR482f, expression of its both putative target genes Solyc08g075630 and Solyc08g076000 were not significantly decreased. In contrast, slmiR5300 targets Solyc05g008650 and Solyc09g018220 were greatly suppressed by the presence of the miRNA; in the case of Solyc09g018220 transcript levels were reduced by almost 90% (Fig. 5A).

We investigated possible translational control of target gene expression by checking levels of the target proteins, using western blot analysis with antibody against the FLAG-tag that was placed at the N-terminus of each target in our constructs. Our results showed that protein levels of all targets were down-regulated by the presence of the corresponding miRNA (Fig. 5B). Proteins corresponding to slmiR482f target gene Solyc08g076000 and slmiR5300 target gene Solyc05g008650 were difficult to detect (Fig. 5B). These results strongly suggest that slmiR482f and slmiR5300 are responsible for the down-regulation of their respective protein targets. The observation that levels of Solyc08g075630 and Solyc08g076000 proteins were greatly reduced, while transcript amount was only slightly affected, suggests that slmiR482f silences these two targets mainly via translational inhibition. These results are consistent with the predictions from the psRNATarget algorithm described above. On the other hand, both mRNA and protein levels of Solyc05g008650 and Solyc09g018220 were reduced by their corresponding miRNA, suggesting that slmiR5300 mainly acts at the transcriptional level. The results for Solyc09018220 contrast with the prediction of translational inhibition by psRNATarget. Taken together, these results revealed that all *in silico* predicted target genes are influenced at the mRNA abundance and/or protein level by co-expression of slmiR482f or slmiR5300.

The four miRNAs target cleavage sites were then validated by RNA Ligase-Mediated 5′ Rapid Amplification of cDNA Ends (RACE) [54], [55] using total RNA isolated from the *N. benthamiana* leaves used for the co-expression studies described above. The 5′ end of the 3′ derived cleavage product without enzymatic pretreatment can be ligated directly to an RNA adaptor with T4 RNA ligase. Gene-specific 5′ RACE primers were designed to yield predicted products of between 300–400 bp if miRNA-guided cleavage occurred *in vivo*. In this way, miRNA-guided cleavage can be detected by sequence analysis of the cloned PCR products. With each primer, a major PCR product of the size predicted to be generated from a template resulting from a miRNA-guided cleavage event was detected. In all cases, at least 80% of the 5′ ends of inserts terminated at a position corresponding to the miRNA (Fig. 5C). With the exception of Solyc05g008650, all of the predicted miRNA-mRNA interactions contain mismatched positions. These findings show that perfect base pairing during the miRNA-mRNA interaction is not a strict requirement to guide cleavage of target RNAs, a result which has been reported by several groups [54], [56].

### Silencing of slmiR482f and slmiR5300 target genes renders the Motelle cultivar susceptible to *F. oxysporum*

We investigated a possible role for the four target proteins in resistance to *F. oxysporum* using a TRV-based virus-induced gene silencing (VIGS) system to down-regulate expression of each gene in the resistant tomato cultivar Motelle. For these studies, *Phytoene Desaturase* (*PDS*) TRV-silenced plants (TRV-PDS) were used as a positive control for silencing. The photobleached phenotype was consistently observed on the third and fourth leaves above the inoculated leaves 3–4 weeks after TRV infiltration [57]. Therefore, treatment with *F. oxysporum* was carried out four weeks after TRV infection.

**Figure 6:**
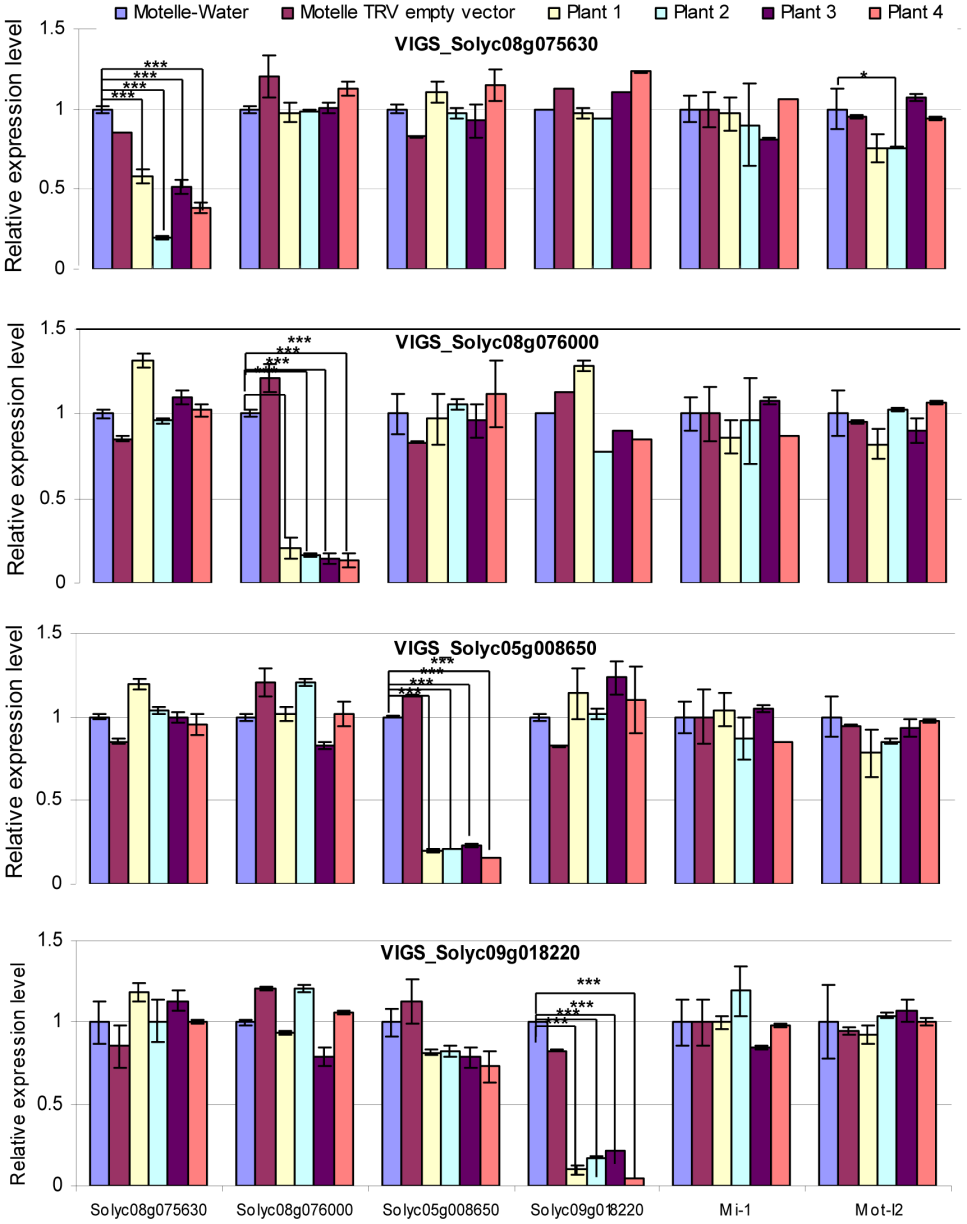
qRT-PCR to assess the degree of gene silencing and to determine possible off-target effects in VIGS plants. Leaflets were harvested four weeks after VIGS and prior to infection with *F. oxysporum*. Total RNA was isolated and subjected to qRT-PCR to evaluate expression of the four predicted miRNA target genes, along with *Mi-1* and Mot-*I-2* controls. The silenced gene is indicated in the title for each horizontal panel, while the gene transcript being measured is shown under the y-axis at the bottom of the figure. Plants treated with water or the TRV empty vector are included as negative controls. mRNA levels are expressed relative to the Motelle plant not treated with TRV for each VIGS construct. Values were normalized using tomato 18S rRNA. Errors are expressed as the standard error. Significant differences are indicated by asterisks. Among all of the VIGS plants, only VIGS Solyc08g075630 Plant#2 exhibits evidence of an off-target reduction in Mot-*I-2* transcript levels relative to the water-treated control. * and *** indicate significant differences when compared to the corresponding control plants in the same treatments at p<0.05 and p<0.001, respectively.

Transcript levels of genes were checked using qRT-PCR prior to *F. oxysporum* infection. All VIGS plants were tested for expression levels of all four miRNA target genes, as well as *Mi-1* and *I-2*, in order to detect possible off-target effects of the VIGS constructs (Fig. 6). The results demonstrated down-regulation of the corresponding mRNA for all four VIGS-target genes, with reductions ranging from ∼60–95% compared to control Motelle plants not treated with a TRV vector (Fig. 6). The VIGS was specific for the silenced genes, with the exception of one VIGS plants (#2) for the Solyc08g075630 gene, which had significantly lower levels of *I-2* expression than the water-treated control. However, the observation that the other three plants were not significantly different is consistent with a specific effect on the Solyc08g075630 gene.

The VIGS constructs for all four target genes corresponded to the extreme 3′ end of the ORF and a portion of the 3′ untranslated region. The VIGS constructs for three out of four target genes did not display nucleotide identity with any other genes in the tomato genome using BLAST. However, the Solyc08g076000 VIGS construct exhibited significant nucleotide identity with a region of tomato gene Solyc02g014230 (38 identical nucleotides in the longest stretch). In order to determine whether the Solyc08g076000 VIGS construct down-regulated expression of Solyc02g014230, we performed qRT-PCR on the same RNA samples used in Fig. 6. The results revealed that Solyc02g014230 mRNA levels were not decreased in the Solyc08g076000 VIGS plants (Fig. S3).

**Figure 7:**
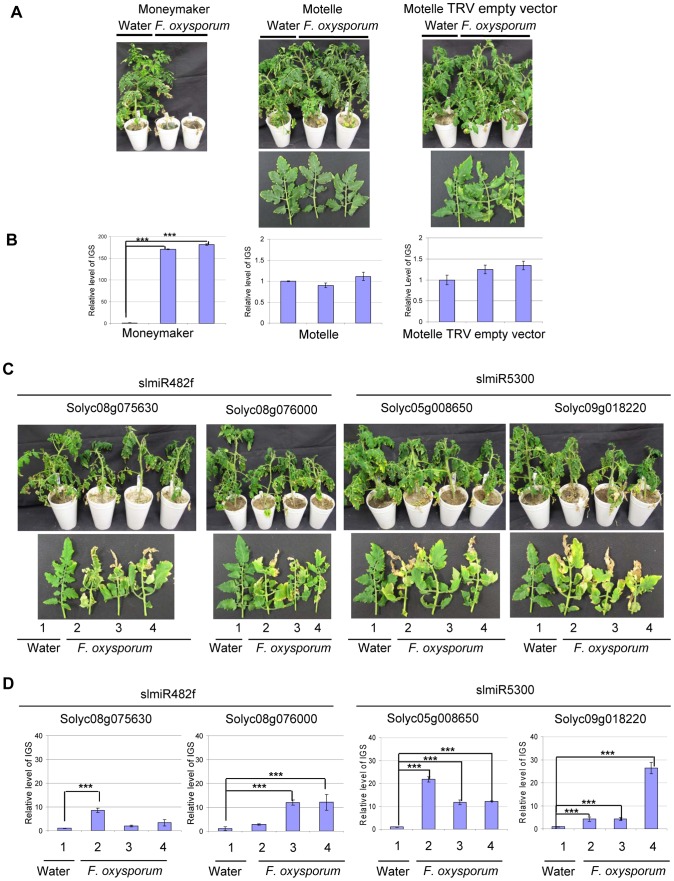
Targets of slmiR482f and slmiR5300 are required for *F. oxysporum* defense in tomato. All control and VIGS plants were cultivated together in the same growth chamber. Similar results were obtained in three biological replicates. Errors are expressed as standard error. *** indicate significant differences when compared to the corresponding control plants in the same treatments at p<0.001. A. **Phenotype of control plants infected by** ***Fusarium oxysporum*** **.** Two-week-old seedlings of the indicated cultivars were treated with water or *F. oxysporum* and photographed four weeks later. Cultivars were Moneymaker (left panel), Motelle (center panel) and Motelle infected with TRV empty vector (right panel). B. **The relative level of** ***F. oxysporum*** **in control plants.** Leaves were collected from water or *F. oxysporum*-treated plants and genomic DNA isolated. qPCR of the Intergenic Spacer region (IGS) of *F. oxysporum* was used to measure relative fungal cell loads *in planta*. Note the different y-axis scale for Moneymaker (left graph) vs. Motelle (center and right graphs). C. **Phenotypes of VIGS plants after infection with** ***F. oxysporum*** **.** Motelle tomato plants were infiltrated with constructs carrying vectors that would lead to VIGS of a predicted target gene. Results for the two targets of slmiR482f are shown to the left, while the two for slmiR5300 are shown to the right. Four weeks after initiation of VIGS, roots of tomato plants were infected with *F. oxysporum* or with water (controls). Phenotypes were scored four weeks later. A water-treated control VIGS plant or a leaf is shown on the left of each panel, with three plants or leaves from *F. oxysporum*-infected VIGS plants to the right. D. **qPCR to determine relative levels of** ***F. oxysporum*** **cells in leaves of VIGS plants.** qPCR of the *F. oxysporum* rRNA intergenic spacer region was performed on genomic DNA isolated from leaves of water or *F. oxysporum*- infected plants as described in (B).

Having demonstrated that the VIGS constructs reduced expression of the appropriate genes in tomato, we assessed disease phenotypes for control and VIGS plants. Scoring was performed four weeks after *F. oxysporum* infection. Control Motelle plants treated with water and VIGS control Motelle plants carrying an empty TRV vector did not exhibit disease symptoms after infection with *F. oxysporum* (Fig. 7A). As expected, Moneymaker plants infected with *F. oxysporum* displayed severe wilting symptoms (Fig. 7A). Disease symptoms, including leaf wilting and discoloration, were observed in all VIGS plants inoculated with *F. oxysporum* but not in water-treated controls (Fig. 7C; water-treated controls are plant or leaf #1 in each panel). All *F. oxysporum*-infected plants carrying a target VIGS construct grew more slowly than control plants treated with water (Fig. 7C) and exhibited wilting at the top leaves. In particular, line 2 of TRV-Solyc05g008650 and line 4 of TRV-Solyc09g018220 exhibited especially severe disease symptoms (Fig. 7C).

We quantified the degree of *F. oxysporum* infection in tomato leaves by amplifying the rRNA Intergenic Spacer Region (IGS) from genomic DNA isolated from leaves using qPCR [58]. In the control plants, levels of *F. oxysporum* were significantly elevated in Moneymaker after infection (Fig. 7B, left panel), while they were relatively unchanged in Motelle with or without TRV vector (Fig. 7B, center and right panels). The extremely high fungal load in Moneymaker after infection with *F. oxysporum* is in agreement with the severe disease symptoms of these plants (Fig. 7A). For plants carrying a VIGS construct, our data indicate that *F. oxysporum* levels were elevated in inoculated plants for each of the four target genes (Fig. 7D). The greatest levels were observed in line 2 of TRV- Solyc05g008650 and line 4 of TRV- Solyc09g018220 (Fig. 7D), consistent with disease severity symptoms. However, the phenotypes observed in VIGS plants were not as severe as those of the control Moneymaker plants after infection (Fig. 7A).

## Discussion

In this study, we exploited the availability of near-isogenic susceptible and resistant cultivars of tomato towards *F. oxysporum* to identify miRNAs important for plant defense. The results with these two cultivars guided our experiments, allowing us to focus on miRNAs that were down-regulated in the resistant Motelle cultivar during infection. We were able to quickly narrow down to a small group of miRNAs and identify two (slmiR482f and slmiR5300) that correlated with disease. Knock-down of the target genes (Solyc08g075630 and Solyc08g076000 for slmiR482f and Solyc05g008650 and Solyc09g018220/*tm-2* for slmiR5300) caused the resistant Motelle cultivar to become susceptible to *F. oxysporum*. Our study provides a platform for differentially expressed miRNAs in tomato after *F. oxysporum* infection and demonstrates that plant miRNAs are involved in defense against *F. oxysporum*.

Due to extensive DNA sequence homology with another gene (Solyc02g014230), we were not able to produce a VIGS construct that was specific for the slmiR482f target Solyc08g076000. However, this construct resulted in susceptibility of the Motelle cultivar to *F. oxysporum* infection, and accumulation of fungal biomass comparable to that observed during knockdown of the other three miRNA target genes. Furthermore, qRT-PCR results demonstrated that Solyc08g076000 mRNA levels were greatly reduced, while Solyc02g014230 levels were relatively unchanged in the VIGS plants. Although we cannot rule out an effect on expression of Solyc02g014230 at an earlier time point that might affect plant defense, this finding supports an active role for Solyc08g076000, but not Solyc02g014230, in resistance to *F. oxysporum* in tomato.

Tomato is one of the most economically important crops and a model system for fruit development. Although whole-genome sequencing of domesticated tomato has made it possible to characterize the entire family of miRNAs, only a small number of miRNAs have been implicated in tomato-specific processes, such as fruit development and ripening [59], [60], [61]. Our study showed that some disease resistance or abiotic stress-associated miRNAs, such as slmiR482f and slmiR398, are suppressed in the resistant tomato cultivar Motelle after *F. oxysporum* treatment. In addition we determined that slmiR5300, for which no functions had been previously ascribed [24], [25], was also suppressed in Motelle during *F. oxysporum* infection. SlmiR482 and slmiR5300 are members of the miR482/2118 superfamily and members of this family have been shown to target the p-loop motif in the mRNA of the NB-LRR encoding *R* genes [23]. The miR482 family has six members, including miR482a-f [23], [45], [46], [47].

Plant defense responses can be activated very rapidly by pathogen infection. Studies in both plant and animal systems have demonstrated that some small RNAs are induced quickly and specifically by various pathogens and diseases [62], [63], [64]. Interestingly, employing the psRNATarget algorithm, the top targets predicted for slmiR482f (Solyc08g075630 and Solyc08g076000) and slmiR5300 (Solyc05g008650 and Solyc09g018220) encode proteins with partial or full NB-domains. Surprisingly, Solyc09g018220 is allelic with the susceptible allele of the ToMV *R* gene *tm-*2. This finding implicates *tm-2* in resistance to fungal attack in tomato and suggests that susceptible disease resistance alleles could have roles in immunity. It is intriguing to speculate that plants are able to use susceptible disease resistance alleles to broaden the pathogen recognition spectrum. Since slmiR482f should also target both the functional *Tm-2^2^* and broken *Tm-2* alleles, it is likely that these genes can also perform the role demonstrated here for *tm-2* in *F. oxysporum* resistance. The discovery of the need for additional full-length or truncated genes for a presumed single gene resistance has broad implication in breeding for resistance and transfer of these traits to plants from different families. It remains to be seen whether the interfamily barrier seen in the transfer of *R* genes is due to the absence of these additional genes [65].

None of the four miRNAs target *I-2* and the three non-*tm-2* targets are not homologs of known R proteins in tomato or other plant species (Fig. S4). Surprisingly, silencing each of their respective genes resulted in susceptibility of Motelle tomato to *F. oxysporum*. Although silencing of Solyc09g018220/*tm-2* resulted in the most severe symptoms, the phenotypes of any single gene VIGS Motelle plant were not as drastic as those observed in the susceptible tomato cultivar Moneymaker lacking *I-2*. This could be because down-regulation of one NB-domain containing protein is not sufficient to completely abolish effective disease resistance in tomato. Alternatively, the residual transcript levels of target genes in VIGS plants may have produced this outcome. Taken together, these results support the requirement for multiple proteins carrying the NB domain, including *tm-2*, in resistance of tomato to *F. oxysporum*.

There are other studies where inadequate expression of resistance to a eukaryotic plant pathogen occurs because resistance genes are suppressed or underexpressed in solanaceous plant species. For example, Tai et al. demonstrated that defense gene expression is relatively reduced in potato cultivars that are tolerant vs. resistant to the fungal pathogen *Verticillium dahliae* [66]. In another study, resistance to the oomycete pathogen *Phytophthora infestans* was analyzed by performing global transcriptional profiling of a susceptible and resistant tomato line using microarrays [67]. The resistance is encoded by a yet unidentified QTL locus. The results showed that VIGS of an *R* gene that was upregulated in the resistant (but not susceptible) line after pathogen infection led to reduced resistance in the normally resistant line. The mode of regulation of the transcripts in these studies was not reported, but the possibility remains that some of the affected mRNAs are down-regulated by miRNAs.

Recently, several examples have been described for the requirement of a pair of NB-LRR proteins for the recognition of a specific Avr and disease resistance in a number of plant species, including Arabidopsis, *N. benthamiana*, rice and wheat [68], [69], [70], [71], [72], [73]. In great majority of these cases, their corresponding *R* genes are located next to each other in tight physical linkage. However, in spite this physical linkage, not all these *R* gene pairs are homologous [69], [73]. All four NB-domain containing sequences identified in our study are distinct from *I-2* and each other. Moreover, all four are located on different chromosomes than *I-2*, suggesting a distinct evolutionary mechanism for the *I-2* resistance. Similarly, the three non-*tm-2* targets do not appear to have a close evolutionary relationship to *tm-2* (Fig. S4). It remains to be determined whether the various targets interact directly with *I-2*, participate in the *I-2* signaling complex or activate parallel signaling pathways [72].

Our results suggest that slmiR5300 catalyzes cleavage of both target mRNAs, while the two slmi482f targets are regulated at the translational level. There is a precedence for regulation of mRNA cleavage by miR482 family members in tomato [23]. We do not know of any examples where a miR482/2118 miRNA superfamily member regulates targets at the translational level. However, Arabidopsis miRNA172 regulates cell-fate specification as a translational repressor of APETALA2 [74] and miRNA156/157 inhibits translation of the SBP box gene, *SPL3* [75]. It is worth noting that miRNAs have been demonstrated to generate secondary siRNAs from the 3′-UTR side of the target RNA sequence, and these secondary siRNAs can regulate gene expression in plants [22], [45], [76], [77], [78]. In particular, slmiR482a, a miR482/2118 superfamily member, targets the *LRR1* mRNA as a siRNA-mediated secondary target [23].

To conclude, our results support the notion that the miR482/2118 superfamily-mediated reduction of gene expression involves multiple NB-domain-encoding genes, including *tm-2*, and occurs via mRNA cleavage and/or translational control mechanisms in tomato. It remains to be determined whether introduction of artificial miRNAs that silence mature and or precursor forms of slmiR482f and slmiR5300 could up-regulate target gene expression in the susceptible Moneymaker plants. In this scenario, we would expect that silencing of miRNAs will enhance resistance to *F. oxysporum* and would therefore be a useful molecular tool to uncover functional roles for the increasing number of discovered miRNAs in tomato.

## Materials and Methods

### Inoculation of tomato plants, small-RNA library construction and deep sequencing

Two tomato near-isogenic cultivars (cv.) Motelle (*I-2/I-2*) and Moneymaker (*i-2/i-2*) that exhibit different susceptibilities to the root pathogen *F. oxysporum* were used for plant infection and library construction. The wild-type *Fusarium oxysporum* f.sp *lycopersici* strain used for all experiments is FGSC 9935 (also referred to as FOL 4287 or NRRL 34936). Profiling experiments were performed on two-week-old tomato seedlings grown at 23°C with a 16/8-h light/dark cycle. Plants were removed from soil and roots incubated in a solution of *F. oxysporum* conidia at a concentration of 1×10^8^/ml for 30 min. Control tomato plants were treated with water. Plants were then replanted in soil and maintained in a growth chamber at 25°C for 24 h with constant light. Plants were removed from soil, and roots rinsed and excised using a razor blade. Roots were immediately frozen in liquid nitrogen and stored at −80°C. Total RNA was isolated from roots using either a method involving hot phenol extraction [79] or Trizol (#15596-018, Qiagen, Grand Island, NY) according to the manufacturer's recommendations. Small RNA libraries for deep sequencing were constructed as described [80] and sequenced using an Illumina GSII sequencer at Los Alamos National Laboratory (Los Alamos, NM).

### Northern blot analysis and quantitative RT-PCR

For small RNA northern blot analysis, 40 µg total tomato root RNA was resolved on 7 M urea/15% denaturing polyacrylamide gels in 1× Tris/Boric Acid/EDTA (TBE). miRNA-specific oligonucleotide probes (Table S3) were end-labeled using γ-32P-ATP (#M0201, New England Biolabs, Ipswich, MA; oligonucleotide probes were labeled according to the manufacturer's recommendations). Blots were stripped and reprobed using a U6 RNA oligonucleotide probe to provide a loading control. All blots were imaged using a PhosphorImager (Molecular Dynamics/GE Life Sciences, Pittsburgh, PA) and band intensities quantified using Imagequant software (GE Life Sciences).

Expression of target or control mRNAs was determined using northern blot analysis or quantitative reverse transcriptase PCR (qRT-PCR). For northern analysis, 20 µg of total RNA was resolved on 1.2% agarose gels and processed as described [81]. Probe templates were prepared by amplification of genomic DNA using specific primers in PCRs (Table S3). This was facilitated by the availability of sequence in the 3′ UTR (which exhibits the greatest diversity between genes, even close homologs) for most genes. In the case of *I-2*, the published sequence was not present in the tomato genome sequence at the Sol Genome database, presumably because the sequenced cultivar lacks *I-2*. Therefore, *I-2* primers had to be designed from a region of the ORF. In order to ensure specificity of amplification, we were able to identify two primer sequences with at least six mismatches with the other genes (Fig. S5). Probes were labeled using the random priming method according to the manufacturer's protocol (#U1100, Promega, San Luis Obispo, CA). Blots were stripped and reprobed using an 18S rRNA probe as a loading control. Blots were imaged and band intensities quantitated as described above for the small RNA northerns.

For qRT-PCR analysis, one µg of total RNA was used for cDNA synthesis (#4368813, Life Technologies, Grand Island, NY) according to the manufacturer's recommendations. Amplification of *S. lycopersicum* miRNA (slmiRNA) targets was carried out using qRT-PCR (iQ5, Bio-Rad, Philadelphia, PA). The sequences of primers used in qRT-PCR are listed in Table S3.

### RNA-seq analysis, normalization of sequence reads and identification of differentially expressed miRNAs

Illumina sequence reads were processed to remove adaptor sequences and quality filtered with FASTX toolkit (Table S1; http://hannonlab.cshl.edu/fastx_toolkit/). Annotation of miRNA gene expression was determined using CLC Bio Genomics Workbench (http://www.clcbio.com/) miRNA mapping pipeline mapping against miRBase 17 (http://www.ncbi.nlm.nih.gov/pubmed/21037258) (Table S2). Further validation of expression for closely related copies was applied using Bowtie alignments of short reads against the miRNA sequences of the *S. lycopersicum* miRNA 482a-f family (Table S4).

To identify differentially expressed miRNAs across *F. oxysporum* infected and H_2_O treated tomato plants, we first mapped the pre-processed sequencing reads to the genomic loci of the known miRNAs (using Bowtie) based on miRBase annotation (version 15, http://www.mirbase.org). In order to deal with noise in sequencing, we only considered miRNAs with at least 12 raw sequencing reads mapped in a given library. In the mapping, we allowed up to 3-nt shifts upstream and downstream from the annotated starting locus of a miRNA to compensate for possible variation in Dicer activities (Table S2).

### Prediction of miRNA target genes

To predict miRNA target genes, we followed the rules for target prediction as described by Allen *et al.* [76]. The psRNATarget algorithm that predicts targets of plant miRNAs [50] was used in this study (http://solgenomics.net). There were several modifications, including no allowance for gaps in the miRNA. A maximum of three continuous mismatches was allowed if the mismatch region contained at least one G∶U pair. The penalty score of the region was multiplied by 1.5.

### Co-expression of miRNAs with predicted mRNA target genes in *Nicotiana benthamiana* leaves

miRNAs and their corresponding target genes were inserted into vector GATEPEG100. All constructs were transformed into *Agrobacterium tumefaciens* strain GV3101. *N. benthamiana* plants, seeded directly in pots, were maintained in an incubator at 24°C with 12 h light/12 h dark cycle. *A. tumefaciens* cultures were grown in liquid LB medium with selection [82]. After 40 h, leaves were harvested, and protein extraction was performed [83]. Proteins were separated on 10% SDS–PAGE gels and transferred onto nitrocellulose membranes (Millipore, Billerica, MA). Membranes were blocked using 5% milk in 1×TBST and then incubated with Anti-FLAG (DYKDDDDK) Antibody (#635691, Clontech, Mountain View, CA) followed by a secondary horseradish peroxidase (HRP)-conjugated goat anti-Rabbit polyclonal antibody (#A0504, Sigma, St. Louis, MO) according to the manufacturer's recommendations. Reactive species were visualized using SuperSignal West Pico Chemiluminescent Substrate (#34087, Pierce, Rockford, IL) and imaging using a Biochemi system (UVP, Upland, CA).

### Validation of miRNA targets

Target validation was done using a RNA ligase-mediated rapid amplification of cDNA ends (5′RACE) assay as described [54], [55], with slight modification using the FirstChoice RLM-RACE Kit (#AM1700, Invitrogen, CA). Total RNA was isolated from *N. benthamiana* leaves used for co-expression of miRNAs with predicted mRNA target genes. Poly(A^+^) mRNA was prepared by two rounds of purification with an Oligotex mRNA Midi Kit (#70042 Qiagen) and directly ligated to the FirstChoice RLM-RACE Kit RNA Oligo adaptor without further modifications. Gene-specific primers were designed approximately 400 nucleotides to the 3′ side of predicted target sites (Table S3). The conditions used for this amplification step were those for gene-specific RACE recommended by the manufacturer.

### Virus-Induced Gene Silencing (VIGS) constructs and *Agrobacterium*-mediated virus infection

VIGS was used to suppress expression of the predicted mRNA targets using TRV-based vectors (pTRV1 and pTRV2) [84]. Gene-specific VIGS constructs could only be developed for three of the four target genes in tomato, due to high nucleotide identity between Solyc08g076000 and Solyc02g014230 (see Results). slmiR482f target genes Solyc08g075630 and Solyc08g076000 and slmiR5300 target genes Solyc05g008650 and Solyc09g018220 were amplified using gene-specific primers (Table S3) and cloned into the pTRV2 vector. A vector carrying a fragment of the *Phytoene Desaturase* (*PDS*) gene was used as a positive control for silencing [57]. All TRV-VIGS constructs were transformed into *A. tumefaciens* strain GV3101. Bacterial cultures were grown as described above. Equal volumes (OD_600_ = 1) of *A. tumefaciens* carrying pTRV1 and suspensions containing pTRV2-derived constructs or pTRV2 empty vector were mixed prior to infiltration into leaves of 2 to 3-week-old tomato plants. pTRV2 empty vector was used as the negative control in this study [57], [84], [85], [86]. Plants were maintained at 20°C for four weeks, until photobleaching symptoms were observed in the leaves of *PDS* TRV-silenced plants. At this time, leaflets were harvested from several plants for isolation of RNA and qRT-PCR analysis of the target genes to assess the degree of silencing. The same plants were then treated with *F. oxysporum* or water as described above for the small RNA library construction. After four more weeks, plants were scored for disease symptoms. Genomic DNA was isolated from leaves [87], [88] and relative levels of *F. oxysporum* determined using qPCR of the rRNA intergenic spacer (IGS) sequence of *F. oxysporum* [58] using specific primer sequences (Table S3).

## Supporting Information

Figure S1 **All predicted targets of slmiRNA482f and slmiRNA5300 in the tomato genome.** Alignments were made using ClustalW2 with slmiRNA482f or slmiRNA5300 and predicted target sequences from the Sol Genomics database (http://solgenomics.net). The nucleotides shown in red in each mRNA target are mismatches with the corresponding miRNA.
(TIF)

Figure S2 **mRNA levels for predicted target genes in tomato cultivars infected by** ***F. oxysporum*** **.** Twenty µg of total root RNA were used for northern blots. Blots were stripped and reprobed using an 18S RNA probe as a loading control. Blots were imaged and bands quantitated as described in Figure 2.
(TIF)

Figure S3 **Expression of Solyc02g014230 is not reduced in VIGS-Solyc08g076000 tomato plants.** Leaves from plants subjected to VIGS using the Solyc08g076000 construct using were harvested three weeks after VIGS. Total RNA was isolated and subjected to qRT-PCR to check expression of the Solyc02g014230 gene.
(TIF)

Figure S4 **Phylogenetic analysis of the** ***I-2*** **gene family and the four miRNA targets.** All protein sequences were obtained from the Sol Genomics database, except for *I-2*, which was taken from reference [28]. Alignment and tree building were performed using MEGA5.2.2 [90].
(TIF)

Figure S5 **DNA sequence alignment of** ***I-2*** **and other genes used for design of** ***I-2*** **-specific primers for qRT-PCR analysis.** ClustalW2 was used to align the DNA sequences of *I-2*, *Mi-1* and sequences homologous to *I-2* in the Sol Genomics database. The regions of *I-2* used to design 5′ and 3′ primers for qRT-PCR are indicated with pink shading. There is a minimum of 6/20 (5′ primer) or 7/21 (3′ primer) mismatches when comparing primers for *I-2* and the other genes in the alignment.
(TIF)

Table S1 **MicroRNAs identified using deep sequencing.**
(XLSX)

Table S2 **MicroRNAs with at least one raw sequence read in at least one library.**
(XLS)

Table S3 **Primers used in this study.**
(DOC)

Table S4 **Sequence reads for members of the miRNA482 a-f family.**
(XLSX)
