# Supplementary figures and images for: MicroRNAs Suppress NB Domain Genes in Tomato That Confer Resistance to Fusarium oxysporum
Source: PLoS Pathog. 2014 Oct 16;10(10):e1004464. doi: 10.1371/journal.ppat.1004464 (PMC4199772; doi:10.1371/journal.ppat.1004464)

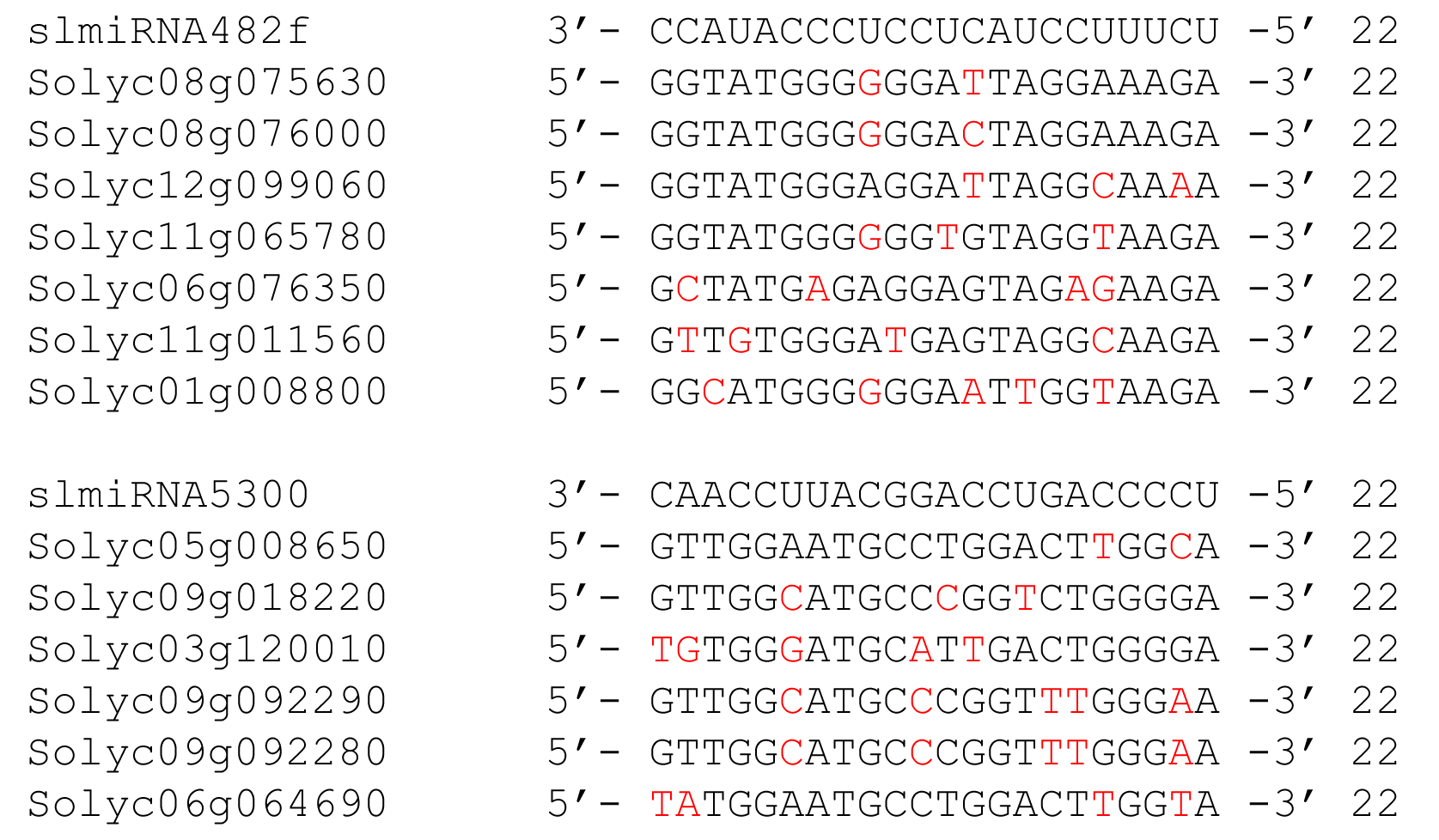

Supplement: Figure S1 — All predicted targets of slmiRNA482f and slmiRNA5300 in the tomato genome. Alignments were made using ClustalW2 with slmiRNA482f or slmiRNA5300 and predicted target sequences from the Sol Genomics database (http://solgenomics.net). The nucleotides shown in red in each mRNA target are mismatches with the corresponding miRNA. (TIF) [file ppat.1004464.s001.tif]

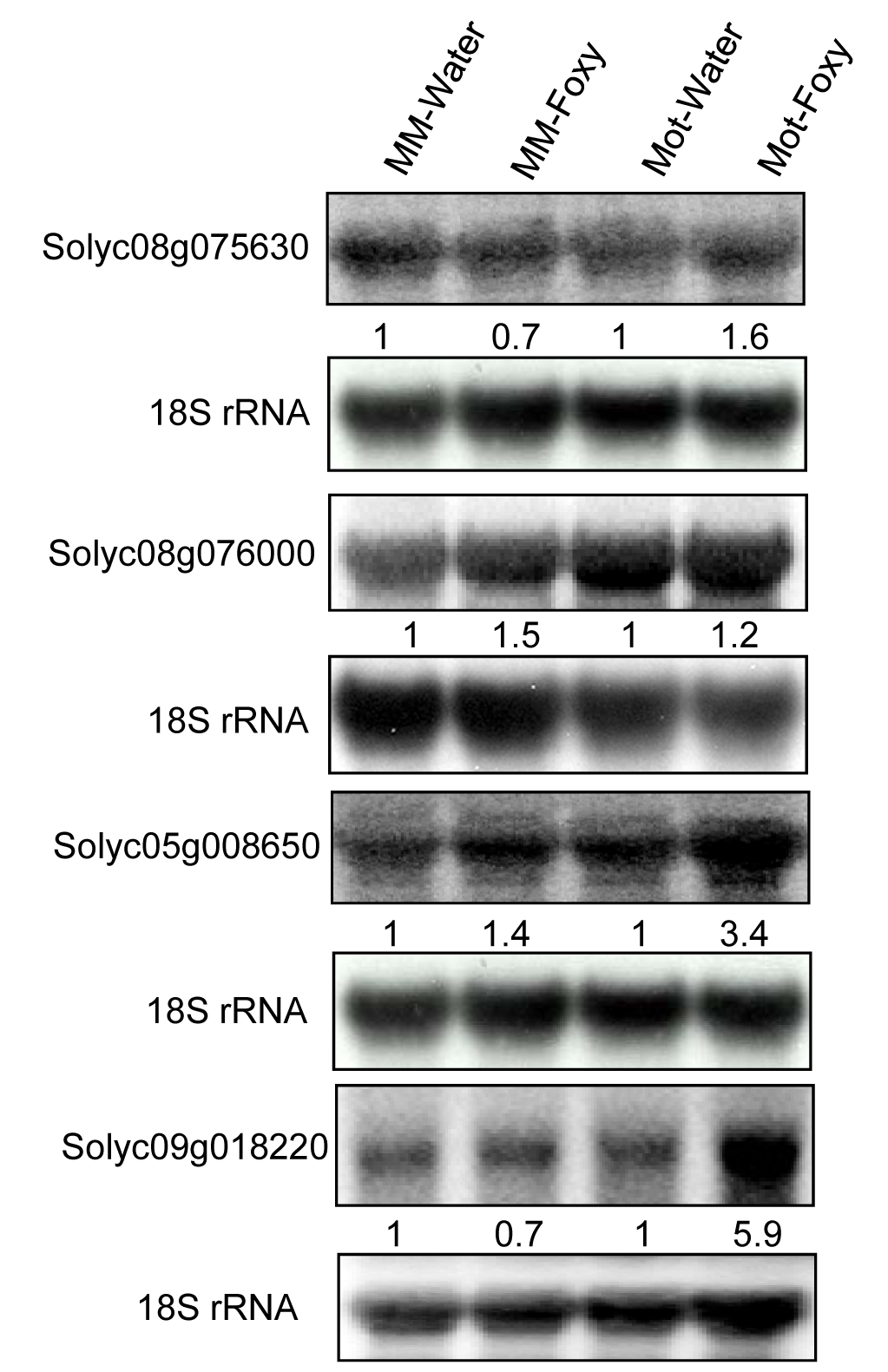

Supplement: Figure S2 — mRNA levels for predicted target genes in tomato cultivars infected by F. oxysporum . Twenty µg of total root RNA were used for northern blots. Blots were stripped and reprobed using an 18S RNA probe as a loading control. Blots were imaged and bands quantitated as described in Figure 2. (TIF) [file ppat.1004464.s002.tif]

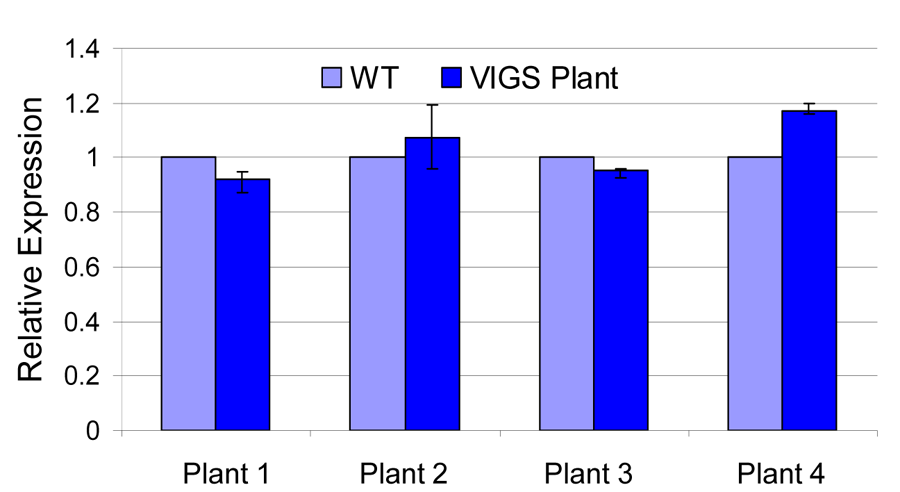

Supplement: Figure S3 — Expression of Solyc02g014230 is not reduced in VIGS-Solyc08g076000 tomato plants. Leaves from plants subjected to VIGS using the Solyc08g076000 construct using were harvested three weeks after VIGS. Total RNA was isolated and subjected to qRT-PCR to check expression of the Solyc02g014230 gene. (TIF) [file ppat.1004464.s003.tif]

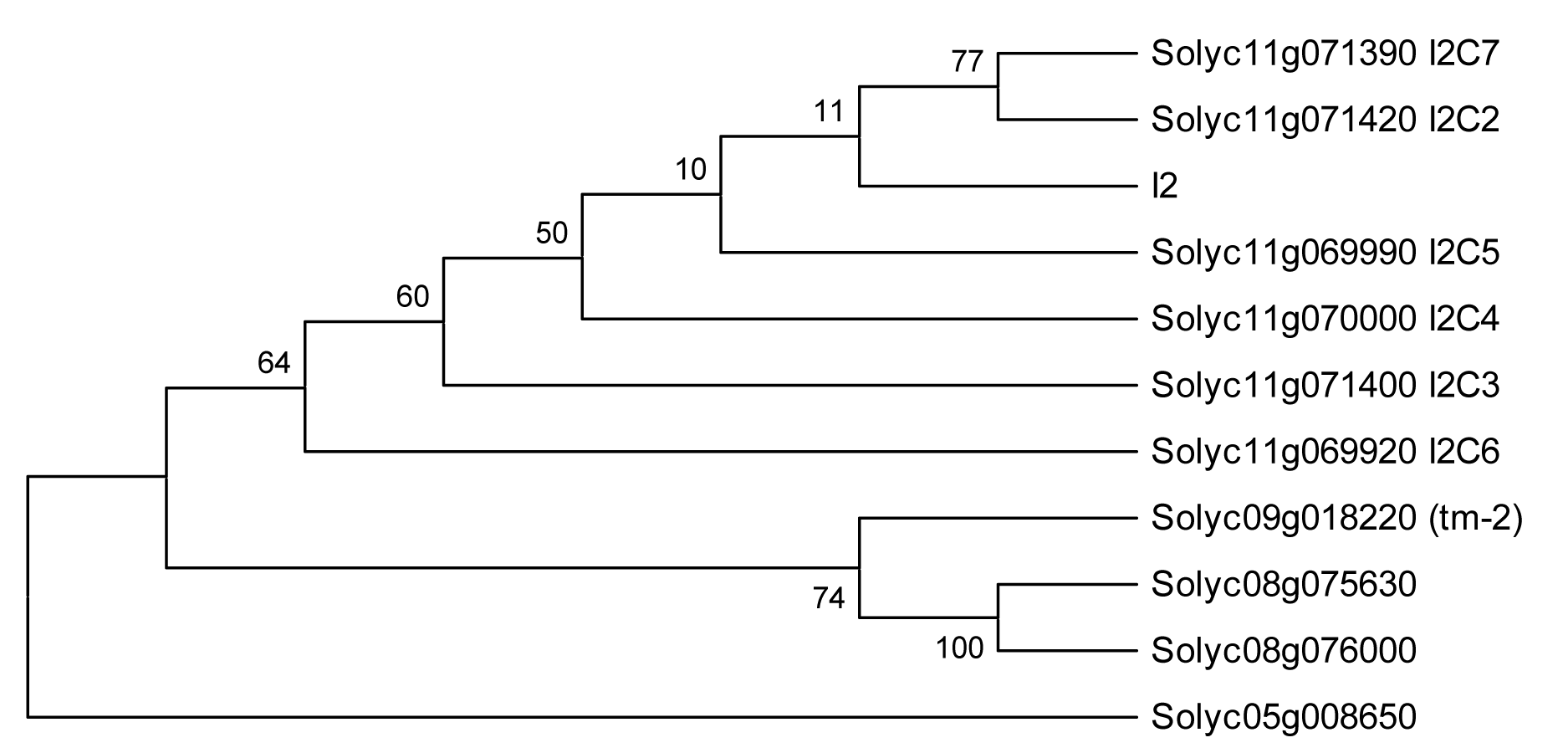

Supplement: Figure S4 — Phylogenetic analysis of the I-2 gene family and the four miRNA targets. All protein sequences were obtained from the Sol Genomics database, except for I-2, which was taken from reference [28]. Alignment and tree building were performed using MEGA5.2.2 [90]. (TIF) [file ppat.1004464.s004.tif]

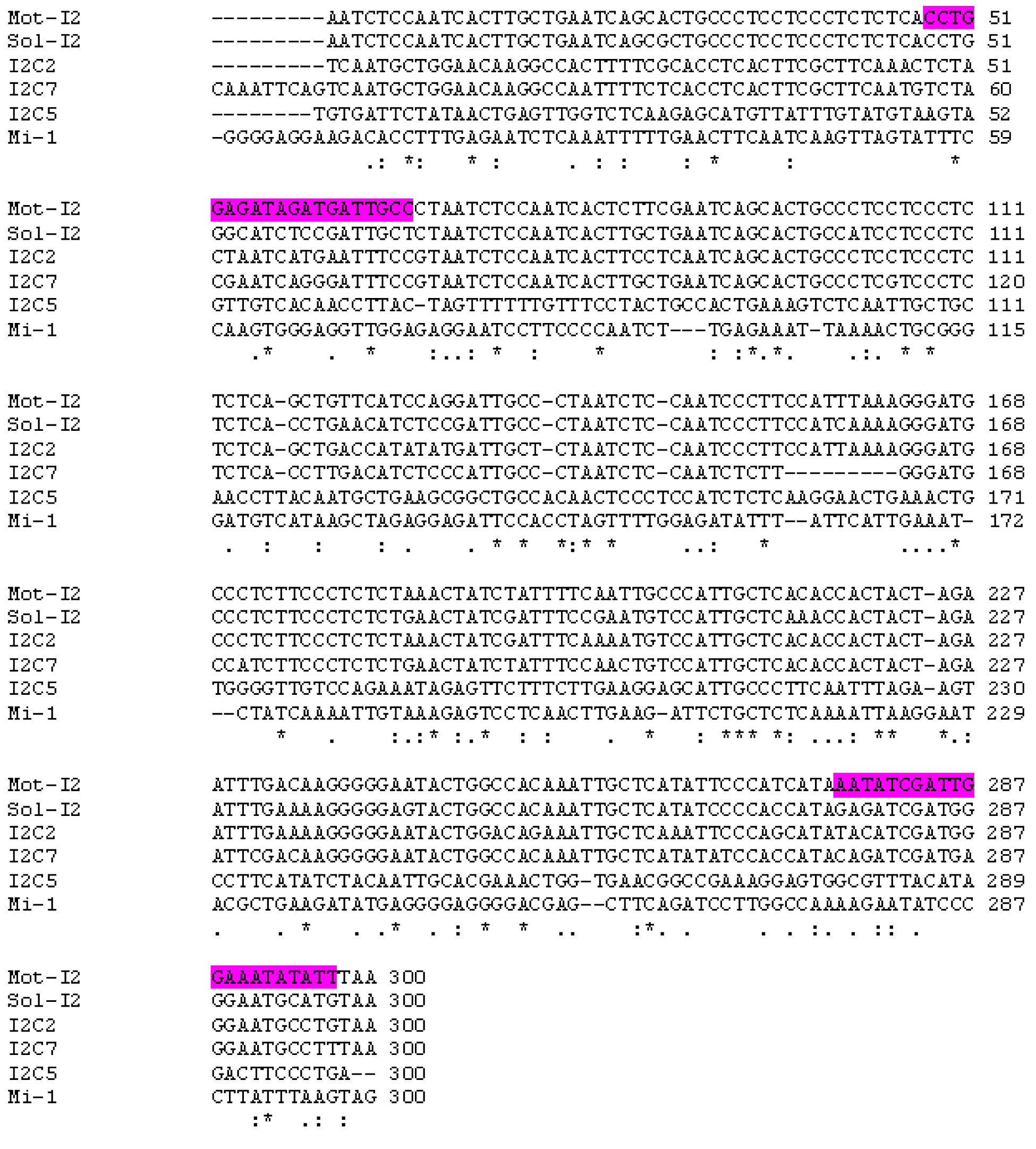

Supplement: Figure S5 — DNA sequence alignment of I-2 and other genes used for design of I-2 -specific primers for qRT-PCR analysis. ClustalW2 was used to align the DNA sequences of I-2, Mi-1 and sequences homologous to I-2 in the Sol Genomics database. The regions of I-2 used to design 5′ and 3′ primers for qRT-PCR are indicated with pink shading. There is a minimum of 6/20 (5′ primer) or 7/21 (3′ primer) mismatches when comparing primers for I-2 and the other genes in the alignment. (TIF) [file ppat.1004464.s005.tif]
