# Supplementary material for: MicroRNAs Suppress NB Domain Genes in Tomato That Confer Resistance to Fusarium oxysporum
Source: PLoS Pathog. 2014 Oct 16;10(10):e1004464. doi: 10.1371/journal.ppat.1004464 (PMC4199772; doi:10.1371/journal.ppat.1004464)
Supplement: Table S3 — Primers used in this study. (DOC) [file ppat.1004464.s008.doc]

**Table S3. Primers used in this study**

| **Primer Name** | **Application** | **Sequence (5’ – 3’)** |
| --- | --- | --- |
| slmiR162 | **MicroRNA Northern Blot Probe** | TGGATGCAGAGGTTTATCGA |
| slmiR403 | ” | CGAGTTTGTGCGTGAATCTAA |
| slmiR162a | ” | CTGGATGCAGAGGTTTATCGA |
| slmiR396a | ” | CAGTTCAAGAAAGCTGTGGAA |
| slmiR167d | ” | CCAGATCATGCTGGCAGCTTCA |
| slmiR394a | ” | GGAGGTGGACAGAATGCCAA |
| slmiR5300 | ” | GTTGGAATGCCTGGACTGGGGA |
| slmiR482f | ” | GGTATGGGAGGAGTAGGAAAGA |
| slmiR157d | ” | GTGCTCTCTATCTTCTGTCA |
| slmiR166g | ” | GAGGAATGAAGCCTGGTCCGA |
| slmiR156 | ” | TGTGCTCACTCTCTTCTGTCA |
| slmiR530 | ” | TAGGTGCAGGTGCAAATGCA |
| slmiR5304 | ” | GGGATGAGTATGTAGCATTGA |
| slmiR398b | ” | CAGGGGCGACCTGAGAACACA |
| slmiR482c | ” | GGTAGGGGAGGAGTAGGAAAGA |
| slmiR482e | ” | GGCAGGGGAGGAGTAGGAAAGA |
| slmiR482d | ” | GGGATGGGAGGAGTAGGAAAGA |
| slmiR482a | ” | GGCATGGGCGGTGTAGGCAAGA |
| slmiR482b | ” | GGATGGGGCGGTATTGGCAAGA |
| U6 | ” | GGGGCCATGCTAATCTTCTCTG |
| q Solyc08g075630-F | **q-PCR Primer** | ACTTGACAGCTACTTCGCTC |
| q Solyc08g075630-R | ” | TTGTTAGGTGCTGCAATCCC |
| q Solyc08g076000-F | ” | ACTCTTACTTCCCTCCGCAT |
| q Solyc08g076000-R | ” | CCTTATCACAGCGCTTTTCC |
| q Solyc05g008650-F | ” | GGTGTGGCATATGAAGTGGA |
| q Solyc05g008650-R | ” | TGGCTTTTCAGTTTGCTGTG |
| q Solyc09g018220-F | ” | TTTCAGTGATCCGAGTGAGC |
| q Solyc09g018220-R | ” | AAGCACTGCCAGTAGACAAC |
| qMi-1-qPCR-FW | ” | ggaatacgctgaagatatgagg |
| qMi-1-qPCR-RV | ” | gcagaaactttgagaaatccac |
| qMot-I2-qPCR-FW | ” | CCTGGAGATAGATGATTGCC |
| qMot-I2-qPCR-RV | ” | AATATATTTCCAATCGATATT |
| qI2-qPCR-FW | ” | GTACTGGCCACAAATTGCTC |
| qI2-qPCR-RV | ” | TTGATGATGAGCTAGCACGG |
| qI2C2-qPCR-FW | ” | TCCATTGCTCACACCACTAC |
| qI2C2-qPCR-RV | ” | GAAACTGTGAACGCAAAGGG |
| qI2C5-qPCR-FW | ” | TTGCACGAAACTGGTGAACG |
| qI2C5-qPCR-RV | ” | TTGATGATGAGCTAGCACGG |
| qI2C7-qPCR-FW | ” | ATACTGGCCACAAATTGCTC |
| qI2C7-qPCR-RV | ” | ACTTGTGATTGTTATCCCTG |
| 18S-rRNA-F | ” | TGACGGAGAATTAGGGTTCG |
| 18S-rRNA-R | ” | CCTCCAATGGATCCTCGTTA |
| qOMP1049 | ” | TGCGATTTGGACGAGATATGTG |
| qOMP1050 | ” | ATTTGCCTACCCTGTACCTACC |
| n Solyc08g075630-F | **mRNA Northern Blot Probe Amplification** | GGTGTGAGAAGGGAATAGGA |
| n Solyc08g075630-R | ” | CGGAAGAATCAGCAAGTGTG |
| n Solyc08g076000-F | ” | GGAAATAGGAGAAGACTGGC |
| n Solyc08g076000-R | ” | ACAGGGAATTGGTGAAGCTC |
| n Solyc05g008650-F | ” | TGTTGGAATGCCTGGACTTG |
| n Solyc05g008650-R | ” | GAGCCACATCCTCTAAGCTC |
| n Solyc09g018220-F | ” | ACATGCACGGATGATGTCAG |
| n Solyc09g018220-R | ” | GAGACGTGTTCTAATGAGC |
| Solyc08g075630-attB1 | **VIGS Vector Construction** | GGGGACAAGTTTGTACAAAAAAGCAGGCTCATCTGCTTATTACTAATGAGATG |
| Solyc08g075630-attB2 | ” | GGGGACCACTTTGTACAAGAAAGCTGGGTCGGAAGAATCAGCAAGTGTGTTGC |
| Solyc08g076000-attB1 | ” | GGGGACAAGTTTGTACAAAAAAGCAGGCTGGAAATAGGAGAAGACTGGCACAA |
| Solyc08g076000-attB2 | ” | GGGGACCACTTTGTACAAGAAAGCTGGGTTAGTACAGGGAATTGGTGAAGCTC |
| Solyc05g008650-attB1 | ” | GGGGACAAGTTTGTACAAAAAAGCAGGCTTGTTGGAATGCCTGGACTTGGCAA |
| Solyc05g008650-attB2 | ” | GGGGACCACTTTGTACAAGAAAGCTGGGTCAGGTAAGTGCTTGTAACTCAGCTC |
| Solyc09g018220-attB1 | ” | GGGGACAAGTTTGTACAAAAAAGCAGGCTACATGCACGGATGATGTCAG |
| Solyc09g018220-attB2 | ” | GGGGACCACTTTGTACAAGAAAGCTGGGTGAGACGTGTTCTAATGAGC |
| c slmiR166a-FW | **Co-expression Vector Construction** | TTAAGAATTCGTTGAAGTCAAGCTAAGATGCG |
| c slmiR166a-RV | ” | TTAAACTAGTCGAAACAAGTTTTAGTAGGTGCC |
| c slmiR482f-FW | ” | TTAAGAATTCTAGGCTACTAAACACACCCG |
| c slmiR482f-RV | ” | TTAAACTAGTGATTCAATGGCCAAAGCAC |
| c slmiR5300-FW | ” | TTAAGAATTCGAGGTGGCAATATGACAAGG |
| c slmiR5300-RV | ” | TTAAACTAGTATCACTCGCACCAAATGAGC |
| c Solyc08g075630-FW | ” | TTAAGAATTCATGGCTGAAGCTTTCCTTCAA |
| c Solyc08g075630-RV | ” | TTAAACTAGTTTACATCTCATTAGTAATAAGCAG |
| c Solyc08g076000-FW |  | TTAAGAATTCATGGCGGAAGCTTTTCTTCAAG |
| c Solyc08g076000-RV | ” | TTAAACTAGTCTAATGAATATCCAGATTTGG |
| c Solyc05g008650-FW | ” | TTAAGAATTCATGGAGCTAGCTGATAAAGAGTC |
| c Solyc05g008650-RV | ” | TTAAACTAGTTCAAAGTGATTGGTAGCTTTGCAC |
| c Solyc09g018220-FW | ” | TTAAGAATTCATGGCTGAAATTCTTCTTACATC |
| c Solyc09g018220-RV | ” | TTAAACTAGTTCATTTACTCAGCTTTTTAAGCCG |
| Solyc08g075630  5’ RACE-OUTER | **Validation of miRNA Targets** | CCAAATGCACGTTGCATGAACA |
| Solyc08g075630  5’ RACE-INNER | ” | GGGTAGTGGTTAGAACATAAGC |
| Solyc08g075630  5’ RACE-FOR | ” | CCTTGCCCAAATGGTCTTCA |
| Solyc08g076000  5’ RACE-OUTER | ” | CGTAAAAGACCACCAAGAGTC |
| Solyc08g076000  5’ RACE-INNER | ” | CGACGTTCCCATAATTGATCC |
| Solyc08g076000  5’ RACE-FOR | ” | GTGACTAAGCATTTCGATCCC |
| Solyc05g008650  5’ RACE-OUTER | ” | GAGCCACATCCTCTAAGCTC |
| Solyc05g008650  5’ RACE-INNER | ” | GGTAAGTGCTTGTAACTCAGC |
| Solyc05g008650  5’ RACE-FOR | ” | AACTACTGTGGCCAAGAAAC |
| Solyc09g018220  5’ RACE-OUTER | ” | TAGCGGTATACCTCCACATC |
| Solyc09g018220  5’ RACE-INNER | ” | GATTCTAGGGGTTGCAATGC |
| Solyc09g018220  5’ RACE-FOR | ” | GTCTACGTTTCACAACAGCC |
